# Strigolactones, from Plants to Human Health: Achievements and Challenges

**DOI:** 10.3390/molecules26154579

**Published:** 2021-07-29

**Authors:** Valentina Dell’Oste, Francesca Spyrakis, Cristina Prandi

**Affiliations:** 1 Department of Public Health and Pediatric Science, University of Turin, via Santena 9, 10126 Turin, Italy; 2 Department of Drug Science and Technology, University of Turin, via P. Giuria 9, 10125 Turin, Italy; 3 Department of Chemistry, University of Turin, via P. Giuria 7, 10125 Turin, Italy

**Keywords:** strigolactones, Strigol, anti-cancer, antimicrobials, sustainable agriculture

## Abstract

Strigolactones (SLs) are a class of sesquiterpenoid plant hormones that play a role in the response of plants to various biotic and abiotic stresses. When released into the rhizosphere, they are perceived by both beneficial symbiotic mycorrhizal fungi and parasitic plants. Due to their multiple roles, SLs are potentially interesting agricultural targets. Indeed, the use of SLs as agrochemicals can favor sustainable agriculture via multiple mechanisms, including shaping root architecture, promoting ideal branching, stimulating nutrient assimilation, controlling parasitic weeds, mitigating drought and enhancing mycorrhization. Moreover, over the last few years, a number of studies have shed light onto the effects exerted by SLs on human cells and on their possible applications in medicine. For example, SLs have been demonstrated to play a key role in the control of pathways related to apoptosis and inflammation. The elucidation of the molecular mechanisms behind their action has inspired further investigations into their effects on human cells and their possible uses as anti-cancer and antimicrobial agents.

## 1. Introduction

**Figure 1:**
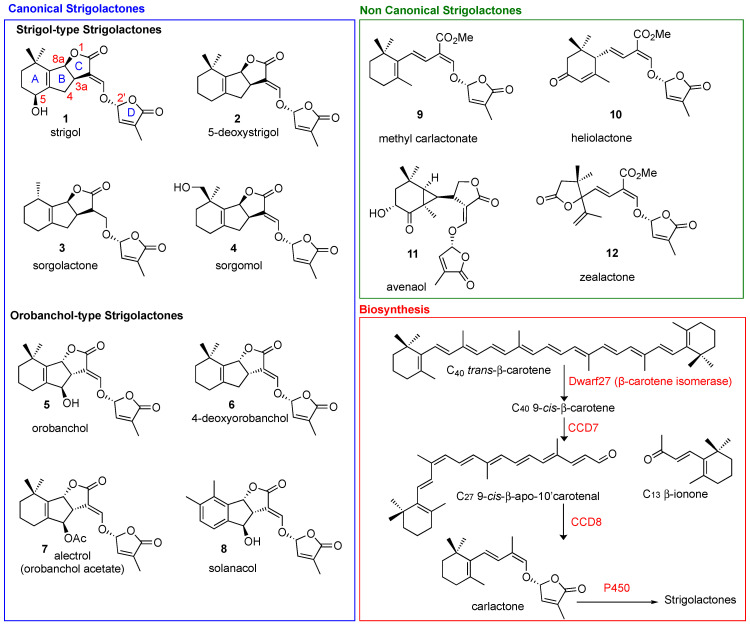
Structural diversity of natural canonical (blue box) and non-canonical SLs (green box). Biosynthesis in red box.

Strigolactones (SLs) are carotenoid-derived sesquiterpene lactones whose structure is characterized by a four-ring system that is generally identified as an ABC tricyclic core linked to a fourth ring, named the D-ring, by means of an enol-ether bridge (Figure 1). The partial elucidation of their biosynthesis in several plant species has identified the involvement of the following genes: DWARF27 (D27; β-carotene isomerase), Carotenoid Cleavage Dioxygenase 7 and 8 (CCD7 and CCD8), and MAX1 homologs (cytochrome P450s) [1]. The first SL, Strigol, was isolated from cotton-root exudate in 1966 [2]; it took over 40 years for its activity as a hyphal branching inducer to be uncovered [3], and for the role of SLs as a new class of phytohormones to be assessed [4,5]. Since then, the boom in interest in the use of these challenging molecules in sustainable agricultural practices indicates that there will be promising forthcoming developments [6,7,8]. Anti-cancer activity has been reported for multiple classes of plant hormones, including cytokinins, methyl jasmonate and brassinosteroids [9], and the first report on the antiproliferative activity of SLs was published in 2012 [10]. An ever-increasing number of references on the exploitation of SLs in the biomedical field have subsequently appeared in the literature. This review highlights the prospects of these future opportunities by outlining the accumulated knowledge on SLs and their potential applications in human health.

## 2. Structure and Synthesis of SLs

### 2.1. Naturally Occurring SLs

According to recent reviews, more than 25 SLs have been identified across the plant kingdom, with different plant species usually exuding different blends of several SLs [11].

Natural SLs are classified into two main classes: canonical and non-canonical SLs (Figure 1), according to the presence or absence, respectively, of the complete ABC-ring system [12,13]. The D-ring and the enol-ether bridge, which acts as a connection to the ABC core of the molecule, are a conserved feature in both canonical and non-canonical SLs. The structural variations in SLs are reflected in their functional diversity [14]. Stereochemistry plays a crucial role in the fine tuning of the biological properties ascribed to SLs [14,15]. Naturally occurring SLs can be divided into two families, strigol- (3aR,8aS in Strigol, Figure 1) and orobanchol-type SLs, (3aS,8aR in Orobanchol, Figure 1), depending on different orientations of the B/C junction, while the D-ring is always R configured (Figure 1). In biosynthetic pathways, the AB-rings can be modified via demethylation, hydroxylation, epoxidation and acetoxylation [16], giving rise to the structural diversification present in natural SLs.

### 2.2. SL Analogs and Mimics

Once the potential applications of SLs in agriculture and biomedicine became striking, synthetic SLs turned out to be an important tool with which to elucidate the functions of these signaling molecules and, at the same time, foster research in the field. Chemical synthesis involves either a total synthesis of the entire SL structure, or the synthesis of analogues with simplified structures that retain SL bio-properties [6,17,18].

**Figure 2:**
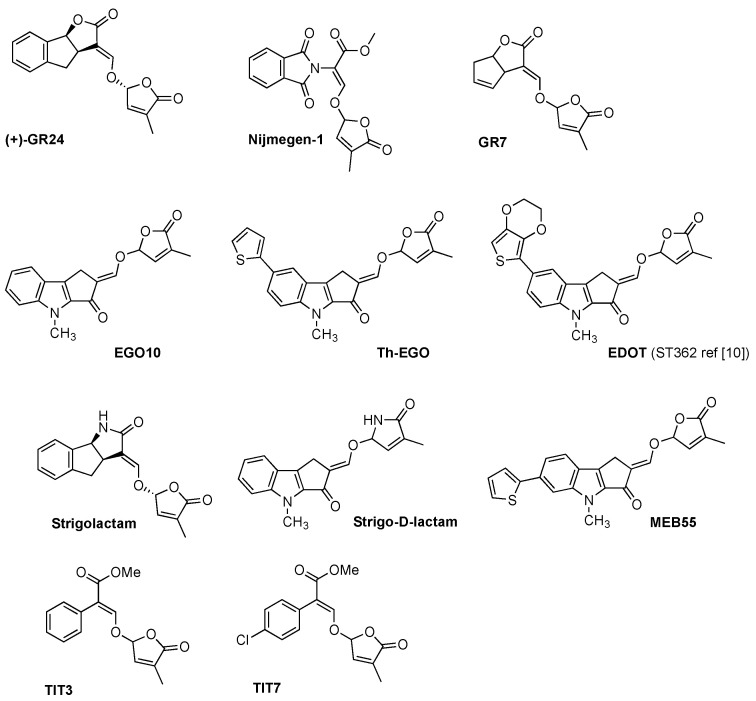
Panel of synthetic strigolactones. (+)-GR24 [6], Nijmegen-1 [19], GR7 [18], EGO10 [20], TH-EGO [20], EDOT [20], Strigolactam [21], Strigo-D-lactam [22], MEB 55 [10], TIT3 and TIT7 [28].

The synthesis of SL analogues is based on the identification of the bioactiphore in SLs. This is the D-ring and the enol-ether bridge connecting C and D-ring (see Strigol **1** in Figure 1), which are required for activity, apparently as a Michael acceptor. Stereochemistry at the D-ring often plays a crucial role, with the most active derivatives showing the same configuration as natural SLs (2′R). A selection of the huge number of synthetic SLs that have been produced so far is provided in Figure 2 and includes GR24 [6], Nijmegen-1 [19], as well as indole derivatives EGO10, TH-EGO and EDOT [20]. Reports have shown that the structural modification of the D-ring into a c-lactam functional group may provide insight into the variations in SL-binding interactions with their receptor [21,22]. Other important analogues are fluorescent SLs, which can be used to track SL perception and trafficking, and include the fluorescence turn-on probe Yoshimulactone Green [23,24,25,26,27]. All these synthetic SLs have greatly contributed to improving our understanding of the biological role of SLs.

## 3. Roles of SLs in Plant Biology

After the first SL was isolated from the root parasite plant *Striga lutea* (witchweed) for use as a germination stimulant, many other SLs, with similar functions, were identified in *Striga* spp., broomrapes, *Alectrs* spp., and other host and non-host plants [2,29,30,31].

All SLs derive from carlactone (CL), which is synthesized in plasmids from all-trans-β-carotene by three different enzymes D27 [32,33], CCD7 and CCD8 [34]. In particular D27, an iron-binding enzyme, catalyzes the isomerization of all-trans-β-carotene to 9-cys-β-carotene, CCD7 converts that to 9-cys-β-apo-10′-carotenal and CCD8 then converts the carotenal into (Z)-(R)-carlactone (CL). The latter is then oxidized by cytochrome P450 monooxygenase MAX1, or other homologous enzymes, to generate the different SLs (Figure 1) [4,5,35]. Interestingly, the genes responsible for SL biosynthesis have been identified in several plants, algae and bryophytes, which suggests that SLs are fundamental molecules that have been maintained by evolution for a very long time [35]. When produced, SLs accumulate in the roots, the main storage organ [36], and then leave them by exudation to reach the rhizosphere, where they can exert their signaling activity [37]. This transport and exudation are regulated by the PhPDR1 transporter, whose mutants have shown a highly reduced level of SLs in root exudate [38].

The biological receptor through which SLs exert their action in plants has been identified as the α/β hydrolase receptor DWARF14 (D14), which is responsible for both the perception and deactivation of hormone signals [39,40]. D14 was first identified in a rice SL-insensitive mutant, but orthologs were soon found in Arabidopsis, petunia and pea [41,42,43]. This enzyme possesses a typical hydrolase catalytic triad, i.e., Ser, His, Asp, and cleaves SLs into the ABC- and the D-ring by performing a nucleophilic attack. The subject has been widely debated [42,44] but, recently, Seto et al., have demonstrated that the active signaling is activated by intact SLs. Upon SL binding, the receptor undergoes a transient conformational adjustment by which it becomes catalytically inactive, but able to interact with D53/SMXLs and D3/MAX2 signaling partners. When the latter are degraded by ubiquitination, the catalytic triad is reconstructed, SLs are hydrolyzed and the signal transduction is interrupted. Thus, the signaling process is triggered by intact SLs and not by hydrolysis or intermediate products [40].

SLs play a number of different roles, which will be explained in more detail hereafter: (i) they control the architecture of above-ground and underground plant organs [4,45,46,47]; (ii) they induce germination in root parasitic plants in genera such as *Striga*, *Orobanche*, *Alectra* and *Phelipanche* spp. [48], which have limited seed reserves, no photosynthetic activity and represent a real threat for agriculture thanks to SL-mediated activation; (iii) they regulate the symbiosis between plants and arbuscular mycorrhizal fungi (AMF); and (iv) they maintain plant life under hostile ecological conditions [35].

SLs are fundamental to controlling plant growth and architecture. Indeed, they induce root growth and the elongation of root hair, but inhibit secondary shoot branching [49]. They also participate, with auxins, in regulating leaf senescence, stem growth and seed germination [50,51,52]. Other phytohormones, such as abscisic acid (ABA), seem to positively regulate SL biosynthesis [53], while SLs behave antagonistically with respect to cytokinins [54], thus underlining the complex interplay of phytohormones that guarantees proper plant behavior. This integrative pathway also sustains the plant response to stress conditions [55]. SL production is, in fact, also regulated by nutrient starvation, such as salt stress, water stress, temperature and nutrient stress conditions. In the case of water stress, for instance, SLs inhibit shoot growth, but stimulate lateral root growth to increase water uptake from the soil. In the case of nutrient stress, the higher amount of produced SLs leads to shoot-branching suppression and stimulates symbiosis with AMF [4]. This latter can, in fact, guarantee the necessary water, phosphate and nitrogen supply, through hyphal extensions. Interestingly, phosphate starvation, as well as AMF colonization, GR24 treatment and naphthylacetic acid, induces the expression of the PhPDR1 transporter [37].

Similarly, in nitrogen-limited conditions, the expression of SL biosynthesis genes is boosted [56]. Moreover, SLs have been proven to respond to biotic stress [57] caused, in particular, by *Rhodococcus fascians*, *Pectobacterium carotovorum* and *Pseudomonas syringae* [58], whose infection induces the upregulation of genes associated to SL production, such as max1, max3 and max4 [59]. However, no alteration was detected in infections caused by bacteria such as *Pythium irregulare* and *Fusarium oxysporum*, thus suggesting that SLs only take part in plant immune response when stimulated by specific bacteria and fungi [35].

## 4. SLs for Sustainable Agriculture: The First Translation

The application of SLs in sustainable agriculture is a challenging goal, but one that is supported by the widespread use in agriculture of technologies that are based on plant hormones to control crop development [60].

Promising results have been achieved with SLs and SL analogs in agriculture, both when they are used as agrochemicals and via developing crop varieties modified for SL production and signaling [61,62].

**Figure 3:**
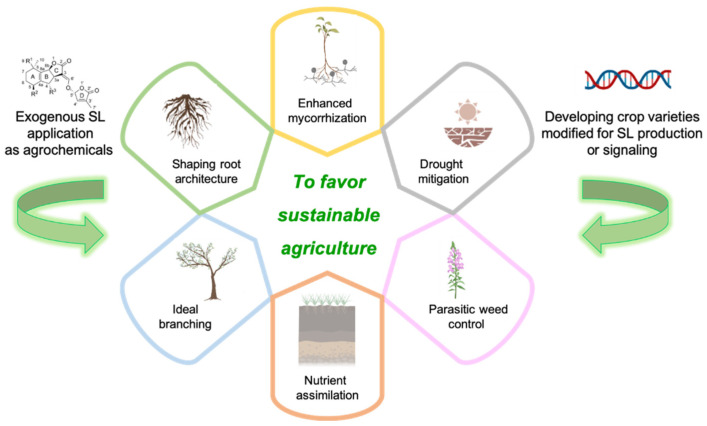
Agricultural applications of SLs. Exogenous applications of SLs as agrochemicals and the development of crops with modified SL production or signaling have the potential to favor sustainable agriculture via a number of mechanisms: shaping root architecture, promoting ideal branching, stimulating nutrient assimilation, controlling parasitic weeds, mitigating drought and enhancing mycorrhization. Created with BioRender.com.

The core components of sustainable agriculture strategies in which SLs display their main application domains are: (i) the control of parasitic weeds; (ii) drought mitigation; (iii) the efficiency of nutrient assimilation and crop development (Figure 3).

### 4.1. SLs in the Control of Parasitic Weeds

One of the most thoroughly investigated applications of SLs is the control of the dangerous parasitic weeds species *Striga* (witchweeds) and *Orobanche* (broomrapes), which are estimated to infest upwards of 60 million hectares of farmland worldwide, resulting in severe yield losses every year [63].

Indeed, crops that produce significantly fewer SLs are more resistant to *Striga* and/or *Orobanche* infection than other cultivars. This has been observed for different species, including rice [64], tomato (*Solanum* *lycopersicum*) [65], the faba bean (*Vicia faba*) [66,67], and pea (*Pisum sativum*) [68]. However, the complete loss of SL exudation is not desirable since it can affect some symbiotic mycorrhizal associations, which are particularly needed in soils that are profoundly affected by *Striga* infestations.

The finding that different SLs have different properties towards mycorrhizae and parasitic weeds has allowed *Striga*-resistant varieties with normal mycorrhization to be obtained. For example, sorghum species mutated at the Low Germination Stimulant 1 (LGS1) locus are resistant to *Striga hermonthica* and *Striga asiatica*, and this resistance can be attributed to a change in profile from strigol-type to orobanchol-type SLs [69]. In field trials, a yield increase in sorghum [70], and maize [71], has been observed in farms across sub-Saharan Africa, where *Striga*-resistant crops were combined with other control measures, such as fertilization and the procedure of non-host trap crops.

When used as agrochemicals, SL analogs have proven themselves to be a realistic opportunity for controlling *Striga* and *Orobanche* via suicidal seed germination. This approach entails the application of SL analogs to soil, followed by the induced germination of the parasites, which cannot survive without the host, thereby depleting the seed bank in the soil. For example, the carbamate SL mimic T-010 reduced *S. hermonthica* emergence by 94–100% in pots and by 33% in sorghum, and is associated with 187–241% increases in sorghum dry weight [72]. Similarly, the SL analogs Nijmegen-1 and Nijmegen-1 Me were effective in controlling *Orobanche ramosa* in tobacco (*Nicotiana tabacum*) crops [73], while a novel class of SLs analogues, derived from dihydroflavonoids, exhibited higher potential in the suicidal germination of the Broomrapes, even compared to the control GR24 [74]. Another approach for the prevention of parasitic seed germination is to antagonize SL responses using SL receptor inhibitors, such as triazole ureas, as agrochemicals [75].

### 4.2. SLs in Drought Mitigation

Another challenging application of SLs is in the improvement of drought tolerance and decreasing yield losses that are caused by adverse climate conditions that lead to low water availability and high salinity [55,76]

It has been observed that water deprivation increase the expression of SL biosynthesis genes in *Arabidopsis* leaves [55], tomato shoots [76], and rice [77]. Interestingly, rice root extracts exhibited increased SL content under water deprivation, and the expression of the genes involved in SL biosynthesis was increased in both the roots and shoots of different species, such as in the crown of tall fescue (*Festuca arundinacea*) [78,79]. By contrast, osmotic stress represses SL biosynthesis in tomato [76], and *Lotus japonicus* roots [80].

A number of observations have highlighted that the foliar application of GR24, a synthetic SL analog, in SL mutants of *Arabidopsis* *thaliana* or grape, can lessen the effects of drought [55,81].

The underlying molecular mechanism is still to be understood, but data are available about the capability of SLs to promote stomata closure to reduce transpiration-associated water loss by interacting with ABA [78,82,83].

Another possibility, in addition to the examples of SL agrochemicals for drought, is the development of drought-tolerant crop varieties via the upregulation of SL signaling. For example, transgenic rice that overexpresses the OsNAC14 transcription factor was observed to upregulate SL biosynthesis genes as well as other genes involved in plant defense, stress response and DNA-damage repair. These transgenic plants had a better survival rate and chlorophyll fluorescence under drought conditions than non-transgenic controls [84].

### 4.3. SLs in the Promotion of Nutrient Assimilation and Crop Development

SLs can also be optimized to improve nutrient assimilation, thus favoring crop enhancement [85,86].

For example, there is evidence to demonstrate that both natural and synthetic SLs (e.g., GR24) endorse plant growth by positively influencing root vigor in different species [47,49].

One interesting strategy is the shaping of the root microbiome by recruiting specific beneficial microorganisms, such as arbuscular mycorrhizal (AM) fungi that promote hyphal branching [87], spore germination, mitochondrial biogenesis and respiration [88], and the exudation of oligosaccharide and protein signals required for AM recognition by the host [89,90,91]. Interestingly, plants mutated for the petunia hybrida ABC transporter (PDR1), a cellular SL exporter with a key role in regulating the development of AM and axillary branches, displayed reduced symbiotic interactions at the root level, indicating that SLs are critical for the establishment of an appropriate root microbiome [38]. A plant’s genetic background influences the degree of mycorrhization and is a key factor in crop success in low-phosphate soils, as confirmed by experiments on SL transporter overexpression, which led to faster mycorrhization in *M. truncatula* [92].

This is a critical point as it supports the idea that SLs play a role in the adaptation of root architecture to variable nutrient accessibility in the soil, mainly nitrogen or phosphorus.

Results obtained from field trials have shown that SL analogs increased the capability of maize and sunflower to efficiently uptake nitrogen, when few fertilizers and pesticides are added [93], and of zucchini squash (*Cucurbita pepo*) [94] and “Hamlin” sweet oranges (*Citrus sinensis*) [95] to do the same under normal growth conditions.

SLs are also involved in legume nodulation processes, and thereby play an important role in nitrogen acquisition. For instance, the application of GR24 increased nodulation in alfalfa (*Medicago sativa*) [96], pea [97], and soybean (*Glycine max*) [98]; conversely, fewer nodules have been observed in SL-biosynthesis mutants than in wild-type plants in *L. japonicus*, pea and soybean [97,99,100,101].

SLs are increased by nutrient stress, such as low phosphate, nitrogen and sulfur conditions [102,103]. For example, the reduction of phosphate levels induces SLs in different families, including cereals, legumes and nightshades [102].

From a different perspective, crop yield can also be increased upon the reduction of SL biosynthesis or perception. For example, genetic approaches applied to modify a rice allele in order to alter SL signaling lead to an improvement in rice architecture. In particular, the analysis of 147 rice accessions identified the CCD7 gene as causing the partial loss of SL-biosynthesis function. Interestingly, CCD7 is widely co-selected with gibberellin deficiency in rice and contributed to improving grain yields during the green revolution [104]. This observation was further confirmed by the detection of an inverse correlation between different levels of tillering across commercial rice cultivars and SL levels [105], as well as by increased tillering in the Nipponbare background upon the silencing of the CCD7 gene by CRISPR/Cas9 [106].

Even though several “proofs of principle” on the potential application of SL in agriculture are now available, future and continued investments will be crucial for their routine and successful application in agriculture. More data about SL bioavailability and stability in plants and soil are certainly required, and the levels of uptake following application under field conditions should be determined. The fast degradation of natural and synthetic SLs in soil [107], and the limited information on early chemical uptake into seeds are major limits [108]. Finally, more clarity is required in the legislation on the production, commercialization and use of potential future SL-based technologies [7].

The optimization of these aspects could potentially allow SLs to be applied at very low quantities, in a range of 1–10 grams/hectare, with undoubtedly positive implications in terms of costs, environment and human safety [61].

## 5. Potential of SLs in Human Health

In recent years, a few studies have begun to shed light on the effects of plant hormones on human health. Molecules such as ABA, salicylic acid, indole-3-acetic acid (the best-known auxin) and cytokinins, which have been extensively studied as plant regulators, are also produced by and elicit biological activities in human cells and animal models [9,109].

Interestingly, several phytohormones can also be produced by human gut microbes, in addition to dietary intake, and likely influence many physiological pathways, such as glucose homeostasis, inflammatory responses and other cellular processes [110,111].

Some phytohormones affect human diseases, such as diabetes, inflammatory bowel disease and cancers, which are also modulated by the gut microbiota [112]. For instance, previous findings have revealed the beneficial effects of ABA against inflammation-related diseases such as type 2 diabetes (T2D), colitis, atherosclerosis, glioma and depression [111]. Salicylates, on the other hand, have long been appreciated as pharmacological agents [113].

Considering these effects, the use of phytohormones as multifunctional nutraceuticals against inflammation-associated diseases, in particular metabolic syndrome and its diverse comorbid symptoms, has been proposed [112]. Overall, the optimal formulation and dosage for phytohormone supplements are still to be established, although the ABA extract of fig fruit has recently been proposed for sugar control against T2D [114].

In this context, the value of SLs in the medical field is only emerging recently. The following sections outline the main discoveries in the applications of SLs for human health.

### 5.1. Modulation of Inflammation

Apart from being involved in the regulation of plant physiology, phytohormones have also been reported to affect human processes including, among others, cell division, glucose metabolism and inflammation [115]. More than ten years ago, it was observed that specific brassinosteroids improve oral glucose tolerance in mice by decreasing the expression of the gluconeogenic enzymes PEPCK and G6Pase and increasing ACT phosphorylation in the liver and muscles [116]. ABA also improves insulin resistance and has a positive effect on neuroinflammation [117], while gibberellic acid (GA) inhibits the release of proinflammatory interleukins, indicating that a GA-enriched diet may alleviate inflammatory disorders [109].

The representative SL GR24 has also been studied for its possible effects on glucose metabolism, and it was found to upregulate and activate SIRT1, a NAD^+^-dependent deacetylase that plays a key role in glucose homeostasis and energy metabolism, and to enhance insulin signaling, glucose uptake, GLUT4 translocation and mitochondrial biogenesis. It is thus a possible new treatment for insulin resistance in skeletal muscle [118].

Recent studies have reported interesting anti-inflammatory activity for GR24 when tested in vitro and in vivo in RAW263.7 cells and zebrafish larvae, respectively [119]. Two GR24 isomers, in particular, were observed to significantly inhibit the release of the pro-inflammatory mediator NO in lipopolysaccharide (LPS)-stimulated cells, as well as the levels of TNF-α and IL-6, compared to the glucocorticoid dexamethasone. Similarly, the levels of phosphorylated NF-κB p65, IκBα, ERK1/2 and p38 MAPK significantly decreased upon treatment with GR24 isomers in a concentration-dependent manner. Indeed, the suppression of NF-κB and MAPK cascades directly resulted in decreased NO, TNF-α and IL-6 production. Important outcomes in the migration of neutrophils and primitive macrophages in zebrafish injuries were also observed. Apart from widening the many possible roles played by SLs, these results also confirmed the importance of the absolute SL configuration and the unsaturated D-ring, whose absence significantly reduced the aforementioned effects [119].

More recently, the role of SLs in neuroinflammation was studied in more detail when phenotypic screenings were performed on SIM-A9 microglial cell lines treated with a GR24 racemic mixture [120]. Again, a reduction in LPS-induced NO production was observed, and this reduction is comparable to that exerted by 1400W, which is a selective irreversible inhibitor of inducible nitric oxide synthase (iNOS). Both mRNA and iNOS levels, generally elevated in neurodegenerative disorders [121,122], were significantly reduced in a dose-dependent manner. ELISA and Western blot again confirmed the downregulation of the TNF-α gene and the consequent inhibition of TNF-α, known to be involved in the activation of α- and β-secretases, which, in turn, stimulate Aβ deposition and the consequent microglial cytokine storm. The suppression of IL-1β production was been registered. These observations support the potential anti-neuroinflammatory and neuroprotective effects of GR24, and reasonably those of SLs in general, against neurodegenerative disorders and the early events of Alzheimer disease (AD). It was also found that GR24 is able to provide the strong dose-dependent downregulation of COX-2, which is responsible for the production of prostaglandins in inflammatory processes [120]. It has to be noted that a clear correlation exists between COX-2 expression and dementia severity in patients affected by dementia, AD and Parkinson disease [123,124]. Interestingly, the nuclear deposition of LPS-induced NF-κB also decreased 3-fold, while PPARγ protein expression, suppressed by LPS treatment, was restored almost completely. Indeed, it has been reported that PPARγ activation can treat and prevent neurodegenerative diseases, and that PPARγ agonists prevent LPS-induced neuronal death [125]. GR24 has also been proven to increase the accumulation of Nrf2, which is the main transcription factor that controls the expression of several cytoprotective enzymes, in microglia cells. In fact, GR24 treatment induced the increased expression of NADPH quinone dehydrogenase-1 (NQO1) and heme oxygenase-1 (HO-1). GR24 seems to have positive efficacy on BBB endothelial cell permeabilization in reducing the negative effects provided by LPS. In particular, treatment with 20 μM GR24 reduced Evans Blue dye extravasation and increased the expression of tight junction proteins, such as occludins. Overall, Kurt et al., have soundly demonstrated that GR24 promotes the downregulation of proinflammatory genes/proteins and the upregulation of cytoprotective ones in microglia and BBB endothelial cells, thus making it an interesting candidate for the development of new treatments for neurodegenerative and neuroinflammatory diseases [120].

Similar effects were previously reported by the same authors in the treatment of murine RAW macrophages and hepatic Hepa1c1c7 cell lines with GR24 [115]. Having confirmed the potent inhibition exerted by the compound on LPS-induced NO production, molecular docking simulations were performed towards the iNOS enzyme, and confirmed the hypothesis to some extent; better interactions and score values were obtained for GR24 enantiomers compared to the positive control 1400 W [115]. As mentioned above, GR24 has an effect on Nrf2 expression. Nrf2 signaling is regulated by the repressor Kelch-like ECH-associated protein 1 (Keap1), which promotes Nrf2 ubiquitination [126]. The disruption of the Nrf2-Keap1 association allows Nrf2 to translocate within the nucleus and induce the expression of phase II detoxification enzymes. Docking simulations were therefore also performed in the Keap1 crevice bound by the Nrf2 peptide. Again, better poses and interaction energies were obtained compared to the control compounds sulforaphane and curcumin. All these data strongly suggest that there exists a link between the activation of Nrf2 and the increased expression of HO-1 and NQO1 cytoprotective enzymes. Indeed, other phytochemicals, such as resveratrol, carnosol, oroxylin A and epigallocatechin-3-gallate, have been demonstrated to exert their protective role through Nrf2 activation in numerous chronic inflammatory diseases, T2D, neurodegenerative disorders, cancer and cardiovascular diseases [127,128,129].

### 5.2. SLs as Anti-Cancer Agents

Several plant-derived compounds have shown anti-cancer activity. The most famous of these include curcumin, which is able to suppress NF-κB and cause apoptosis, vinblastine, an alkaloid that targets microtubule, and paclitaxel (Taxol), which also acts on microtubules [130]. More recently, phytohormones enlarged this category with brassinosteroids, which cause G1 arrest and apoptosis [131], methyl jasmonate, which depletes ATP in cancer cells through mitochondrial perturbation [9,132] and cytokinins. SL analogues have also demonstrated anti-cancer activity in vitro and in vivo.

The first anti-tumoral effect of SLs in breast cancer cells was reported by Pollock et al., who found that these natural compounds can specifically inhibit proliferation and induce apoptosis in cancer cells, while sparing non-cancer cells [10]. The effect of GR24 was first evaluated on ER+ tumorigenic, ER- metastatic and normal non-neoplastic fibroblasts. Significant growth reduction was observed at 2.5–5 ppm concentrations in both cancer lines, while no significant effect was observed on fibroblasts. GR24 was also observed to inhibit the growth and reduce the viability of MCF-7 tumorigenic cells that propagated as mammospheres in non-adherent growing conditions. Similar positive results were obtained when five synthetic SL analogues were tested. In particular, ST362 and MEB55 (later renamed TH-EGO and EDOT, respectively), which are characterized by an indolyl-based structure with an enol-ether bridge connecting the C and D ring, were found to be the most potent and to exert a non-reversible reduction in cell viability after only four hours. This effect is likely associated to the inhibition of the phosphorylation of p38 MAPK and JNK1/2, which are stress-activated kinases that play a key role in a stress-signaling cascade and are associated with cell-cycle arrest and apoptosis [133,134]. Indeed, the mechanism by which SLs exerts their anti-tumoral activity has been associated to the blockage of cell-cycle progression and the consequent induction of apoptosis. The authors only observed a dose-dependent increase of cells in the G2/M phase in tumorigenic cell lines, while normal fibroblasts did not show sensitivity to SLs in this context. This may be linked to the higher division rate of cancer cells and to the capacity of SLs to target rapidly dividing cells.

ST362 and MEB55 were also tested, alone and in combination with the breast cancer chemotherapy drug paclitaxel, in xenograft models [135]. The administration of MEB55 led to reductions in tumor volume and tumor-growth rate in mice implanted with MDA-MB-231 xenografts. ST632 also showed promising results, comparable to those of paclitaxel. The co-treatment of cancer cell lines with MEB55 and paclitaxel showed a two-fold decrease in MEB55 IC_50_, thus suggesting that the two molecules could have an additive effect. However, fewer promising results were obtained on xenografts, as the tumor volume reduction obtained by the co-administration was not significant with respect to treatment with MEB55 alone. The effect of SLs on microtubule bundling has also been studied, and it was found that the phytohormones might affect microtubule network integrity and, consequently, inhibit the migration of the most invasive breast cancer cell lines [136,137]. This might also have an effect on tumor metastatic character, which is strictly related to cell-migration capability. It is interesting to note that paclitaxel also mainly targets microtubules to exert its potent action.

Having demonstrated the inhibition exerted by SLs in breast cancer cells and breast cancer stem cells, the same authors widened the study to other solid and non-solid cancer cell lines, including prostate, colon, lung, melanoma, osteosarcoma and leukemic cells [138]. They found that SLs, in particular the analogues EG5, EG9c, ST357, ST362 and MEB55, were able to inhibit the growth of the cell lines and to induce a cellular-stress response that turned into cell-cycle arrest and apoptosis in all cases, except fibroblasts. In particular, the authors again reported that SLs were able to arrest the cell cycle at the G2 state. This arrest was primarily associated to the down-regulation of cyclin B1, the Cdc25C protein and mRNA levels, and to the activation of stress signaling, such as the induction of multiple heat shock proteins (HSP) and cytokine [139]. The activation of stress signaling exerted by SLs also affects the stress-induced transcription factor FOXO4, p38 MAPK and JNK1/2, which are again involved in the signaling for cell-cycle arrest [133], and apoptosis [134,140]. Moreover, SLs induce the expression of several pro-apoptotic genes and inhibit the expression of survival factors such as ALDH1, which is a key regulator of stem cell viability and self-renewal.

The two most potent molecules were, again, MEB55 and ST362, which were able to induce apoptosis in all tested cell lines and to specifically reduce the viability of prostate tumor conditionally reprogrammed cells (CRC), in which a significant reduction in cyclin B level and a pronounced stress response (pp38 induction) were observed. Similarly, a stronger apoptotic response was observed in CRC tumor cells than in normal cells. These findings support the potential of SL analogues to induce a significant non-reversible apoptotic response in transformed cells and in patient-derived tumor cells, while having significantly lower toxic effects in normal cells [138]. The two compounds were also proven to induce DNA double-strand breaks (DSBs) and consequently activate DNA damage response in osteosarcoma cells [141]. However, at the same time, SLs downregulate the DNA repair protein RAD51 via ubiquitination and, consequently, also the homology-directed repair (HDR) system, which are possibly associated to resistance towards DNA-damaging chemotherapy and radiotherapy. It follows that RAD51 downregulation may be a useful strategy for restoring and enhancing the effectiveness of cancer chemotherapy [141,142]. Importantly, no DSB or cell death was detected in non-transformed fibroblasts, which once again highlights the potential clinical relevance of these molecules.

SLs analogues were also tested in cell lines of hepatocellular carcinoma (HCC), which is the predominant form of liver cancer and the fifth most common type of cancer in men [143]. Two of the tested compounds, namely TIT3 and TIT7 (Figure 2), showed anti-proliferative effects (cell viability reduction) on HepG2 cells, but had a lower effect on hamster kidney cells (BHK cells). The two compounds were also tested on PC3 prostate cancer and T-cell acute lymphoblastic leukemia cell lines, with dose-dependent cell-viability inhibition being shown. This indicates that the two compounds have a capability to inhibit cell proliferation in both solid and hematological tumors. Interestingly, the compounds showed a minimal inhibitory effect on healthy cells compared to cancer cells. The authors also performed a wound-healing assay on HepG2 cells to check cell migration, which was effectively inhibited by both TIT3 and TIT7. A possible mechanism, as already suggested for SLs, could involve the compounds interfering with the microtubular network, but the exact targets and a detailed mechanism of action that explains compound selectivity is currently difficult to define [141].

Taken together, these results clearly support the anti-cancer effects of SLs, which are emerging as a new possible treatment for advanced prostate cancer and other types of tumors.

### 5.3. SLs with Antimicrobial Activity

**Figure 4:**
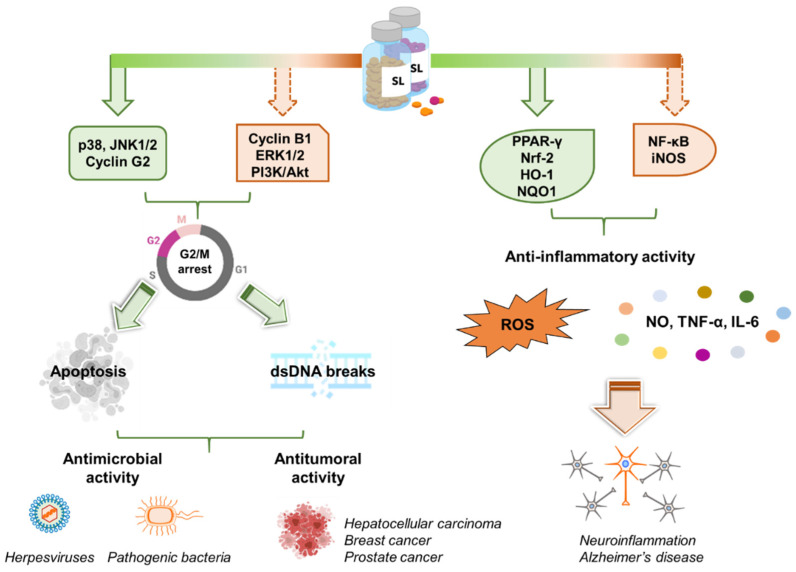
SL activity in human cells and their potential in medicine. *Left panel.* Synthetic analogs of SLs control multiple pathways leading to the arrest of the cell cycle in the G2/M phase. Apoptosis and DSBs are then induced. These properties grant SLs antimicrobial as well as anti-tumoral activity. *Right panel.* SLs exert anti-inflammatory effects by inhibiting the release of inflammatory molecules (e.g., NO, TNF-α, IL-6, ROS). This makes SLs promising scaffolds for the development of novel anti-Alzheimer’s disease candidates. Created with BioRender.com.

Despite the multifaceted roles of SLs in plant biology and their promising features as drug candidates for different kinds of cancers, their antimicrobial and antiviral activity is still unexplored (Figure 4).

The lessons learned from phytopathogenic fungi, in which the SL analog GR24 impairs the growth of root pathogens (e.g., *Fusarium oxysporum f. sp. melonis*, *Fusarium solani f. sp. mango*, *Sclerotinia sclerotiorum* and *Macrophomina phaseolina*), and the foliar pathogens *Alternaria alternata*, *Colletotrichum acutatum* and *Botrytis cinerea* [144], led to the hypothesis that SL antimicrobial activity could be extended to human pathogens.

The possibility of using SLs as antibiotics has been explored for the novel SL analog TIT3 against different pathogenic bacteria. Promising results were obtained for *Staphylococcus aureus*, *Salmonella typhimurium*, *Escherichia coli*, *Klebsiella pneumonia* and *Bacillus subtilis*, indicating that SLs may be a viable alternative for the treatment of different strains of bacteria that are resistant to conventional antibiotics [28].

Recently, our group has demonstrated, for the first time, the efficacy of a group of SL analogues as antivirals against members of the *Herpesviridae* family, in particular human cytomegalovirus (HCMV) [145]. HCMV is a widespread pathogen that can cause severe disease in immunocompromised individuals [146]. In addition, HCMV infection is the most frequent cause of congenital malformation in developed countries [147]. Although nucleoside analogues have been successfully used against HCMV, their use is hampered by the occurrence of serious side effects, the rapid emergence of resistance and the fact that their efficacy is limited to alleviating symptoms, without eradicating the latent infection [148,149]. There is, therefore, an urgent clinical need for new antiviral drugs that can overcome these limitations. Of the different SL analogs screened, there are two compounds that significantly inhibit HCMV replication in vitro, i.e., TH-EGO and EDOT-EGO. These results are challenging in the field of antiviral research, since, besides inhibiting the late phases of the viral cycle, apoptosis has been shown to be a novel strategy that SLs rely on to exert their inhibitory role against viral replication. These results have been confirmed by in-silico molecular docking simulations, which predict a stable protein-ligand complex between the SL analogs and the modeled structure of the putative target IE1, which is employed by HCMV to escape apoptosis [145].

In this context, further investigations on physiologically relevant targets for HCMV infection, such as endothelial and epithelial cells, and on cells that do not progress to a lytic infection, such as monocytes, will be crucial to corroborating and expanding the data obtained on HFFs. Furthermore, it will be essential to extend the analysis to other HCMV proteins, such as other antiapoptotic HCMV proteins (vMIA, cICA, UL38 and IE2), as well as to other members of the *Herpesviridae* family and emerging viruses, for which medical demand is an absolute priority at this time.

## 6. Conclusions

SLs are versatile and challenging molecules. In this review, we have demonstrated how the blooming and interdisciplinary research on SLs continuously unveils exciting, new biological functions and properties for these molecules. The exploitation of these properties is not without challenges: (i) lead compounds with unbiased activity and uncontroversial benefits should be identified; (ii) the synthesis of SLs is complicated, and designing the proper structure to emphasize specific activity is a difficult task; (iii) it is necessary to find the right balance between the stability and reactivity of SLs and effective formulations must be set up. We can foresee that the deep and full understanding of the molecular mechanisms, the elucidation of the transduction signal pathways and the development of their synthetic chemistry will pave the way for a variety of potential applications in agriculture and medicine.

## 7. Patents

Patent “Strigolattoni per uso nella prevenzione e/o trattamento di infezioni da virus della famiglia Herpesviridae” (No: 102018000010142, PCT/IB2019/059611, E7527/19-EW, University of Turin, Italy).
